# The Effect of Storage Temperature and Time on the Concentrations of Bovine Serum Amyloid A and Its Mammary Associated Isoform

**DOI:** 10.1155/2012/861458

**Published:** 2012-03-19

**Authors:** Csilla Tóthová, Oskar Nagy, Herbert Seidel, Gabriel Kováč

**Affiliations:** ^1^Clinic for Ruminants, University of Veterinary Medicine and Pharmacy in Košice, Komenského 73, 041 81 Košice, Slovakia; ^2^Clinic for Swine, University of Veterinary Medicine and Pharmacy in Košice, Komenského 73, 041 81 Košice, Slovakia

## Abstract

The objective of this study was to evaluate the effect of storage under various conditions on the concentrations of major bovine acute phase protein—serum amyloid A, and its mammary isoform. Blood samples were taken from seven clinically healthy calves, and milk samples from six clinically healthy dairy cows. The harvested blood serum and the milk samples were fractioned into aliquots. One aliquot was analyzed on the day of collection without storage. The second aliquots were stored at 4°C for 1 day, the remaining aliquots were kept frozen at −18°C for 2, 7, 14, and 21 days, and then analyzed. Blood serum was analyzed for serum amyloid A (SAA). The concentrations of mammary isoform of SAA (M-SAA) were measured in milk samples. Over time, the concentrations of SAA in serum showed a tendency of significant decrease during storage at −18°C (*P* < 0.01). Similarly, the values of M-SAA decreased significantly in samples maintained at freezer temperatures (*P* < 0.001). In the refrigerated samples, we found non-significantly lower values of SAA, as well as M-SAA. Presented results indicate that the storage temperature and duration may markedly affect the concentrations of bovine SAA and M-SAA.

## 1. Introduction

Biochemical investigations have central function in clinical chemistry with an important aid to provide principal biochemical information for clinicians in the diagnosis, prognosis, monitoring, and screening of diseases. It is widely accepted that the majority of medical decisions are made using laboratory data [1]. Such information will be of value only if it is accurate and relevant and if its significance is appreciated by the clinician so that it can be used appropriately to guide clinical decision-making [2]. The specimen for analysis must be collected and transported to the laboratory according to a specified procedure, and every step in the process of sample handling and analysis requires careful attention if the data are to be of clinical value [3]. In some cases, the analysis is delayed, or the specimens are sent to distant laboratories for analysis. Moreover, the planning for multiple experimental time points often results in samples that will be analyzed together at a later date and thus subjected to different periods of storage before analysis [4]. In such cases, degradation of labile analytes must be prevented by refrigerating or freezing the samples. A lot of authors stated that not only physiological conditions and factors (e.g., age, sex, pregnancy, and nutritional status) but also inadequate biological sample storage, as a potential source of preanalytical errors, may markedly affect the concentrations of many biochemical variables [5–7]. However, the influence of sample storage on the concentrations of less frequently measured biochemical variables, including acute phase proteins, and their stability during storage in veterinary medicine is less well documented.

Acute phase proteins are a group of blood proteins that change in concentration in animals subjected to external or internal stimuli such as tissue injury, inflammation, impaired homeostasis, trauma, or stress [8]. In cattle, serum amyloid A (SAA) is one of the diagnostically most important acute phase proteins with an increase in serum concentrations up to 1000 times, as a result of inflammatory response [9]. Serum amyloid A is a small hydrophobic protein that is the precursor of amyloid A protein, the major protein of *α*-amyloid, so it is potentially involved in the pathogenesis of reactive amyloidosis [10]. Several isotypes of SAA were found. Of these, isotypes SAA1 and SAA2 respond to inflammatory stimuli with markedly increased production in the liver [11]. The SAA3 isoform is expressed in nonhepatic tissues during the acute phase response with increases found in lung or mammary gland [12]. The role of mammary-associated isoform of SAA (M-SAA) is related to newborn adaptation to extrauterine life and mammary tissue remodeling in cows with mastitis [13]. Acute phase proteins have been used as biomarkers of diseases for decades in human medicine [14]. However, although the measurement of the concentrations of acute phase proteins can detect or confirm the presence of infection or pathological lesion and it provides useful information for the monitoring of the health status of animals, acute phase proteins have been relatively underutilised in the veterinary medicine. The use of serum amyloid A has been limited due to difficulties in purification and quantification, probably because it is a hydrophobic apolipoprotein that is complexed within serum high-density lipoproteins [15]. In bovine practice, an increased focus on the application of acute phase proteins, including serum amyloid A, has recently been developed. Stability and storage study is one of the most important studies that should be performed during the course of the introduction of the method of the acute phase protein determination.

For this reason, the aim of the present study was to evaluate the effect of storage under various conditions on the concentrations of major bovine acute phase protein-serum amyloid A (SAA) in serum samples of calves. Additionally, the stability of mammary isoform of SAA in milk samples from dairy cows during storage was investigated.

## 2. Material and Methods

### 2.1. Sample Collection

The effect of storage at freezer and refrigerator temperatures on the concentrations of serum amyloid A during storage was investigated in blood samples from seven clinically healthy female calves from a conventional dairy farm. The calves were of a Slovak spotted breed and its crossbreeds at the age of 4–6 months, and their body weight was 85–140 kg. The evaluated animals were housed loosely in larger groups and fed hay and grain with free access to water. Blood samples for the analyses were collected by direct puncture of *v. jugularis* into serum gel separator tubes without anticoagulant. Blood samples were allowed to clot at room temperature and then centrifuged at 3000 g for 30 minutes to separate serum. The harvested blood serum was fractioned into aliquots. One aliquot was analyzed for the concentrations of serum amyloid A (SAA, *μ*g/mL) immediately after the separation without storage, and these results obtained at time 0 were considered as initial concentrations. The second aliquot of the separated serum was stored in refrigerator at 4°C for 1 day and then analyzed. The remaining aliquots were kept frozen at −18°C, and the concentrations of SAA were determined after 2, 7, 14, and 21 days of storage.

For the evaluation of the changes in the concentrations of mammary isoform of SAA (M-SAA, ng/mL) during storage, milk samples from six clinically healthy dairy cows from a conventional dairy farm were included into this study. The cows were of the Slovak spotted breed and its crossbreeds of 3–5 years of age. The evaluated cows were fed twice a day individual feeding rations according to the milk production and had *ad libitum* access to water. Composite milk samples were collected into plastic tubes by hand-stripping. Forestrips were milked out, and then approximately equal parts of milk from each quarter were taken and mixed together. The collected milk samples were fractioned into aliquots. One aliquot was analyzed for the concentrations of M-SAA on the day of sample collection without storage. The second aliquot of milk samples was stored in refrigerator at 4°C for 1 day and then analyzed. The remaining aliquots were stored at −18°C and were analyzed for M-SAA concentrations after 2, 7, 14, and 21 days of storage.

### 2.2. Laboratory Analyses

The serum concentrations of SAA were analyzed by method of sandwich enzyme-linked immunosorbent assay using commercial ELISA kits (Tridelta Development, Ireland) according to the procedure described by the manufacturer. All the samples, including the standards, were tested in duplicate. The optical densities were read on automatic microplate reader Opsys MR (Dynex Technologies, USA) at 450 nm using 630 nm as reference.

The concentrations of M-SAA were determined by ELISA method using commercial diagnostic kits (Tridelta Development, Ireland) according to the method described for SAA, modified by the manufacturer for the determination of M-SAA in milk samples.

### 2.3. Statistical Analyses

Arithmetic means (*x*), standard deviations (SD), and medians for the concentrations of SAA in serum, as well as milk samples, were calculated using descriptive statistical procedures. The effect of time on the concentrations of evaluated variables during the storage of the samples frozen at −18°C was evaluated by repeated measures ANOVA test with the Tukey-Kramer multiple comparisons test to determine which means differ significantly from one other. The paired *t*-test was used for the evaluation of the differences between the initial concentrations of measured parameters and the values quantified in samples stored 1 day at 4°C. All statistical analyses were performed using the programme GraphPad Prism V5.02 (GraphPad Software Inc., California, USA).

## 3. Results

The data referring to the concentrations of SAA in bovine serum, and the values of M-SAA in milk samples during storage expressed as average values, standard deviations, and medians, including the significance of differences between the mean concentrations, are presented in Tables 1 and 2.

**Figure 1:**
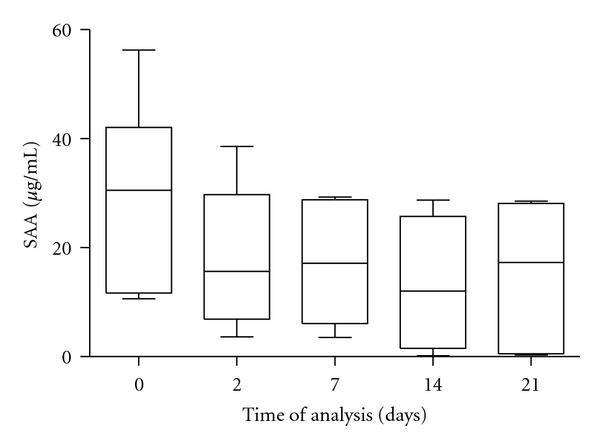
Concentrations of SAA in serum samples during freezer storage. The plots show the median (line within box), 25th and 75th percentiles (box), and minimum and maximum values (whiskers).

The evaluation of the mean serum concentrations of SAA over time showed a tendency of significant decrease of values during storage at −18°C (*P* < 0.01, Table 1). The samples stored in a freezer had significantly reduced concentrations from day 2 onwards (*P* < 0.05), with the significantly lowest concentrations on days 14 and 21 of storage compared with the initial values (*P* < 0.01). More detailed analysis of the serum SAA concentrations showed that 50% of the results of samples without storage (day 0) ranged from 11.70 to 42.10 *μ*g/mL, with median concentration of 30.50 *μ*g/mL (Figure 1). The median SAA concentration recorded on day 2 of storage was about half as lower (15.60 *μ*g/mL) than the initial value, and 50% of measured values were in the range of 6.89–29.70 *μ*g/mL. Similar trend of lower values was found in the next period of freezer storage, with the lowest median concentration of SAA on day 14 of storage (12.00 *μ*g/mL).

**Figure 2:**
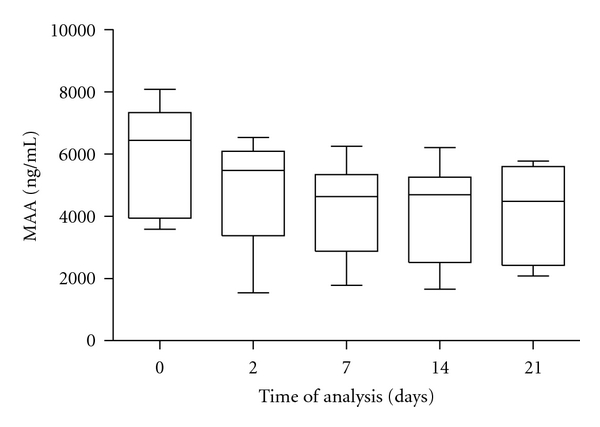
Concentrations of M-SAA in milk samples during freezer storage. The plots show the median (line within box), 25th and 75th percentiles (box), and minimum and maximum values (whiskers).

The changes in the mean concentrations of M-SAA in milk samples in relation to the time of storage at the temperature of −18°C were highly significant (*P* < 0.001, Table 1). For the concentrations of M-SAA in milk samples determined on day 2 of freezer storage, a significant decrease of values was found (*P* < 0.01, Table 1), with further significant decrease of concentrations after day 7 of storage compared with initial values (*P* < 0.001), and then the values remained relatively stable for the evaluated period of freezer storage. In the median concentrations of M-SAA we observed a gradual decrease of values during freezer storage, with the lowest median concentration determined on day 21 of storage (4483.20 ng/mL, Figure 2). By more detailed evaluation of individual M-SAA concentrations we found that during the initial analysis of milk samples 50% of measured values ranged from 3936 to 7326 ng/mL, and in samples analyzed on day 2 of storage we already recorded lower concentrations with the range of 50% of measured values between 3371 and 6091 ng/mL.

The mean concentration of SAA obtained in bovine serum stored at 4°C for 1 day was nonsignificantly lower than the value recorded by the evaluation of samples analyzed immediately (Table 2). Similarly, when analyzing the concentrations of M-SAA in milk samples after the storage in refrigerator, no significant differences were found between the concentrations determined in samples without storage and in milk stored 1 day at 4°C.

## 4. Discussion

Many clinical trials and research studies depend on delayed batch analyses of collected blood samples. To accomplish these analyses, blood samples are separated by centrifugation, and the harvested serum or plasma is quickly frozen [16]. At specific follow-up times, the samples are thawed and analyzed as designated by the study protocol. The stability of some routine clinical biochemistry parameters (total proteins, albumin, lactate dehydrogenase, creatine kinase, trace elements, and hormones) was tested in human and in a range of animal species under different laboratory storage conditions [4, 5, 17]. These studies stated that the temperature and the duration of the storage are important factors, which may impact the results of biochemical analyses. Data from such reports regarding the stability of acute phase proteins and the influence of storage on their concentrations in blood samples are rather scarce. Stability and storage studies were performed to determine the effect of storage on the concentrations of C-reactive protein, as the diagnostically most important acute phase protein in humans [18, 19]. In fact, data on storage stability of acute phase proteins and the effect of the temperature and duration of storage on their concentrations in veterinary medicine are still limited. Because acute phase protein determination may become of importance in laboratory testing also in cattle, for serum amyloid A and its mammary isoform (to be measured correctly), a better understanding of their biological variation and the effect of preanalytical factors on their concentrations is required.

In the present study, we observed a marked effect of sample storage at lower temperatures on the concentrations of serum amyloid A, characterized by different intensities of changes in its concentrations at freezer or refrigerator temperatures. In frozen serum samples, the results showed a trend of significantly decreasing SAA, as well as M-SAA concentrations over time. Significantly lower concentrations were found already after 2 days of storage. The SAA concentrations determined in serum samples stored at refrigerator temperatures differed nonsignificantly from the values measured in samples without storage, but its mean value was lower. Storage stability of SAA was investigated by Hillström et al. [20] in equine serum samples. The authors observed no significant changes in SAA concentrations over time in serum samples stored at 4°C, and the variance between days was not higher than could be explained by the imprecision of the method. Similarly, in another study, where an equine SAA standard pool was stored at 4°C over a period of up to two months, no significant changes in SAA concentrations were noted [21]. These authors observed only a slight fluctuation in measured values that they explained by the imprecision of the method. In addition, no influence on the stability of human SAA and C-reactive protein was found for the samples that were stored at −20°C for 2 months until actual measurement [22]. Solter et al. [23] and Cerón et al. [24] reported that acute phase proteins, including haptoglobin and C-reactive protein, are more stable than the cellular components of blood, and assays can be performed on frozen serum samples. Aziz et al. [18] indicated that the effect of different specimen processing and storage conditions on the concentrations of acute phase proteins may vary depending on the assay configuration and should be validated at the beginning of any research project. According to our findings, bovine SAA appears to be less stable during storage at lower temperatures, predominantly at freezing. Diminished concentrations of SAA at lower temperatures, obtained in our study during freezer, as well as refrigerator storage, may be related to the lability and degradation of this protein resulting from the changes in its molecular configuration during storage. Another reason for variations in the stability of SAA during storage could be its biological behavior [20]. However, the aforementioned contradictory data indicate that further investigations are needed to clarify the possible influence of the temperature and duration of storage on the concentrations of bovine serum amyloid A and to explain the changes in its concentrations during storage at lower temperatures.

The effect of storage temperature and duration on the concentrations of mammary isoform of SAA in milk samples of dairy cows is less well documented. According to the manufacturer data, milk samples collected for the determination of M-SAA can be stored for up to 2 days at 4°C or stored frozen at −20°C for longer periods (Tridelta Development, Ireland). However, to our knowledge, no published studies have been reported on the effect of sample storage on the concentrations of M-SAA in relation to the temperature and duration of storage. In the presented study, we observed a trend of significantly progressively decreasing values of M-SAA during storage at freezer temperatures from day 2 onwards. Similarly, nonsignificantly diminished M-SAA concentrations were found in milk samples maintained at refrigerator temperatures. The aforementioned reduction in the concentrations of M-SAA in milk samples after storage might be caused by degradation of this mammary isoform of SAA, as several factors have been found to affect milk components during storage at various temperatures and time [25]. Woltersdorf et al. [26] reported that the freezing process and the resulting time delay of the analysis may result in physical changes in the sample, which may affect the concentration of the analytes in the sample. The concentrations of acute phase proteins, including M-SAA in milk, are quantified to get information for diagnostic or monitoring purposes [27, 28]. This demands precise measurement of acute phase protein concentrations and analytical stability. According to the presented results, serum and milk samples without freezer storage are recommended for SAA analyses in cattle, including the determination of the concentrations of its mammary associated isoform because the protein degradation during storage at lower temperatures may cause alterations in their concentrations. Seeing that published data on the long-term stability of SAA and predominantly its mammary isoform during storage under various conditions are still limited, further studies may be helpful.

## 5. Conclusions

For analytical approaches proper sample processing and handling is of great importance. Therefore, issues related to the preanalytical factors of acute phase protein measurement should be considered to avoid potential misclassification between sick and healthy animals. Our results indicate that the temperature and duration of storage as a part of proper sample handling are important factors also for some acute phase protein analyses. The results presented in our study showed marked effect of sample storage, predominantly at freezer temperatures, on the concentrations of bovine serum amyloid A and its mammary associated isoform with progressively significant decreasing values over the evaluated time. Thus, after longer lasting storage at lower temperatures, the measurement of SAA, as well as M-SAA, in serum or milk samples may not be accurate, potentially causing erroneous interpretations. According to these results it seems to be important to analyse the samples for the mentioned parameters without storage in freezer.

## Figures and Tables

**Table 1:** The changes in the concentrations of SAA and M-SAA with time during freezer storage.

		Time of analysis (days)					*P*
Parameter		0	2	7	14	21	
SAA (*μ*g/mL)	*x*	30.30^a,A^	17.89^b^	17.42^b^	13.77^B^	13.94^B^	< 0.01
	± SD	17.19	12.32	10.10	10.94	12.07	
	median	30.50	15.60	17.10	12.00	17.30	

M-SAA (ng/mL)	*x*	5945.06^A,x^	4822.77^B^	4261.17^y^	4162.26^y^	4149.15^y^	< 0.001
	± SD	1765.70	1819.70	1559.00	1641.60	1532.60	
	median	6439.60	5471.70	4631.20	4685.90	4483.20	

*P*: significance of ANOVA test.

Means with different superscripts in rows differ significantly at: ^a,b^ *P* < 0.05; ^A, B^ *P* < 0.01; ^x, y^ *P* < 0.001.

**Table 2:** Comparison of the concentrations of SAA and M-SAA analyzed in samples without storage and in samples stored at 4°C for 1 day.

		Samples		*P*
Parameter		Without storage	Stored at 4°C for 1 day	
SAA (*μ*g/mL)	*x*	30.30	26.24	n. s.
	±SD	17.19	14.97	
	Median	30.50	30.80	

M-SAA (ng/mL)	*x*	5945.06	5376.91	n. s.
	±SD	1765.70	5376.91	
	Median	6439.60	5906.90	

*P*: significance of paired *t*-test.
